# Multi-Material Front Contact for 19% Thin Film Solar Cells

**DOI:** 10.3390/ma9020096

**Published:** 2016-02-06

**Authors:** Joop van Deelen, Yasemin Tezsevin, Marco Barink

**Affiliations:** TNO Applied Sciences, High Tech Campus 21, Eindhoven 5656 AE, The Netherlands; y.tezsevin@tue.nl (Y.T.); marcobarink@hetnet.nl (M.B.)

**Keywords:** solar cells, front contact, TCO, metallization, modeling

## Abstract

The trade-off between transmittance and conductivity of the front contact material poses a bottleneck for thin film solar panels. Normally, the front contact material is a metal oxide and the optimal cell configuration and panel efficiency were determined for various band gap materials, representing Cu(In,Ga)Se_2_ (CIGS), CdTe and high band gap perovskites. Supplementing the metal oxide with a metallic copper grid improves the performance of the front contact and aims to increase the efficiency. Various front contact designs with and without a metallic finger grid were calculated with a variation of the transparent conductive oxide (TCO) sheet resistance, scribing area, cell length, and finger dimensions. In addition, the contact resistance and illumination power were also assessed and the optimal thin film solar panel design was determined. Adding a metallic finger grid on a TCO gives a higher solar cell efficiency and this also enables longer cell lengths. However, contact resistance between the metal and the TCO material can reduce the efficiency benefit somewhat.

## 1. Introduction

Photovoltaics (PV) is a wide arena for materials science to demonstrate the power of bringing different materials together in one device. There are two main material based photovoltaic families: one is Si wafer based and the other is thin film PV, which relies on coating of high quality materials on a substrate [1]. Even though thin film PV is based on “simple” coating steps, the solar power conversion efficiency has been improved to values well above 20% and approaches the values previously only reached by Si record cells [2,3]. One of the main drivers behind this success is material improvement. In addition, interface issues have been tackled. For instance, recently back surface passivation in CIGS cells has been improved [4,5]. However, unlike for Si wafer based technologies, the stunning laboratory cell advances have not translated into 20% solar panel efficiencies.

Two of the bottlenecks for thin film solar panels are the active area loss due to interconnection and losses in the transparent front contact, for which usually a transparent conductive oxide (TCO) is coated [6,7]. The loss in active area should be reduced and we detail its impact on overall cell and front contact design in the Results Section. The TCO inevitably has a trade-off between conductivity and transparency [8,9]. In the case of small cells, this hurdle can be masked using small dimensions and addition of a patterned metallic grid to the TCO, thereby compensating for its low conductivity. Such a combination of materials can increase the efficiency by creating a dramatic shift in conductivity at the expense of only a small loss in transmittance [10,11].

Classic wafer based cells do not have a TCO and fully rely on metallic grids. Therefore, the cell layout and ink requirements are highly different from the desired characteristics of monolithically interconnected thin film cells, which requires smaller feature sizes and, in the case of thin film CIGS cells, limited annealing temperatures below 200 °C. A few studies of grid on TCO were performed, but these reflected the status of ink jet printing, resulting in low (<1 µm) and 100 µm wide grids. Because of these low and wide grid dimensions, there was hardly an advantage of this TCO + metal grid approach compared to the TCO only approach [12,13,14,15]. Therefore, in solar panel production, this solution has not been adopted for monolithically interconnected solar cells. 

Recently, however, the full potential of the application of metallic grids with optimized finger and cell dimensions (*i.e.*, lower width and larger height) was reported to give a significant boost in thin film solar efficiencies [16]. Because such approach would add complexity in the manufacturing process, the efficiency gain should be determined and evaluated with respect to manufacturing issues. Previous study [16,17] was performed on cells with efficiencies of 15.5% and many aspects such as cell layout and absorber material band gap, were not discussed in depth. Moreover, previous designs did not take into account the material interface issue of contact resistance. The investigation of contact resistance in solar cells has only briefly been touched [18,19] and its impact on design of monolithically integrated solar panels still needs to be addressed. Moreover, the previous case was limited to low efficiency CIGS or organic PV [15,16] and the case for high efficiency thin film solar cells, spanning a wide range of band gaps should be investigated. In short, there is a lack of knowledge of the impact of the cell layout and specific metal-TCO interaction (e.g., contact resistance) on the preferred grid design and the expected efficiency benefit.

This work focuses on the introduction of metal finger grid to enhance the performance of thin film solar panels with up-to-date cell efficiencies of 19%. The effects of cell length and interconnection area, as well as the band gap of the absorber material and the contact resistance are modeled. In contrast to previous work reflecting a rather idealized situation, specific issues such as the losses due to the specific contact resistance and the impact of reduced irradiation intensity are discussed. The calculated cell efficiencies give guidelines over a wide range of (non-ideal) circumstances for useful front contact technologies that aim to enhance the thin film solar panel efficiency.

## 2. Results and Discussion

### 2.1. General Considerations

**Figure 1:**
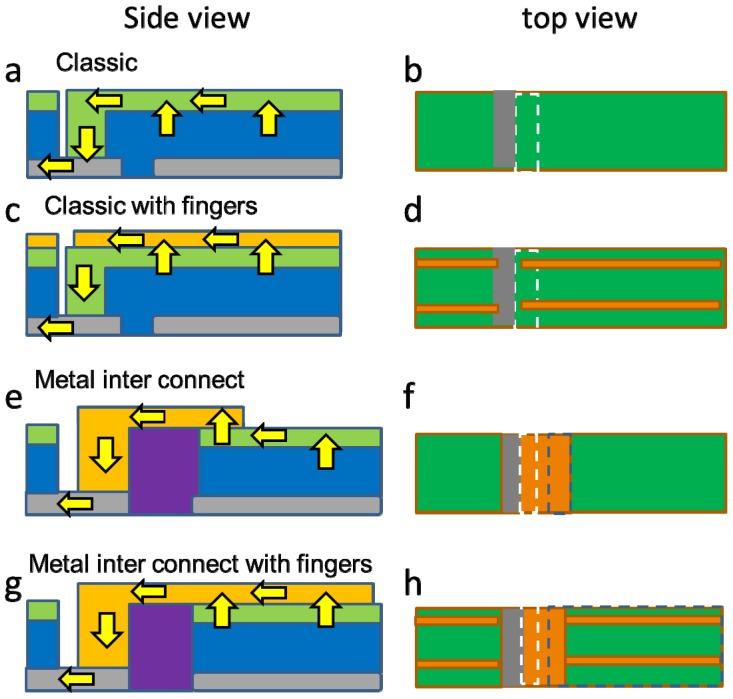
Schematic representations (not to scale) of different interconnection and cell layouts with a side view (**a**,**c**,**e**,**g**) and a top view (**b**,**d**,**f**,**h**). The top image shows the front contact (in green), the absorber material (in blue) and the back contact (in grey). In addition, the separation and interconnection layout between two adjacent cells is shown. The surface area of the TCO/back contact material interface is indicated by the white dashed box. The flow of current is depicted by the arrows. The second highest image shows the case where the front contact is supplemented by a metal grid (in orange), whereas the right image displays the area covered by the metal (not to scale). The third image shows the case of the metal interconnect, for which two material interfaces are important: the metal back contact area represented by the white dashed box and the metal/TCO contact areas represented by the blue dashed box.

In thin film solar panels, the panel is usually divided into parallel cells that are series connected. There are several ways to accomplish this and Figure 1 details two of them.

The first is the classic way, in which the TCO is both the front contact and the interconnect (Figure 1a,b). In this case, the isolation area of the back contact is filled with the semiconducting absorber material and all the current is transported through the TCO. The TCO can be enhanced by a metallic finger grid, while the interconnection between top and bottom electrode takes place at the TCO back contact interface (Figure 1c,d). Alternatively, a metal busbar can be used for this interconnection purpose (Figure 1e,f) [20]. The isolation of the back contact can be filled with a dedicated insulator material [21]. This approach was mentioned to have more design freedom. In addition, the metal can function both as an interconnection and as a top contact enhancer (Figure 1g,h). The contact surface area between the front and the back contact, as indicated by the white dashed box, is not changed by these different layouts. In the case of the metal interconnect, the fingers on the TCO will increase the total contact surface area between the metal and the TCO, which is an important feature, as will be discussed in Section 2.4.

**Figure 2:**
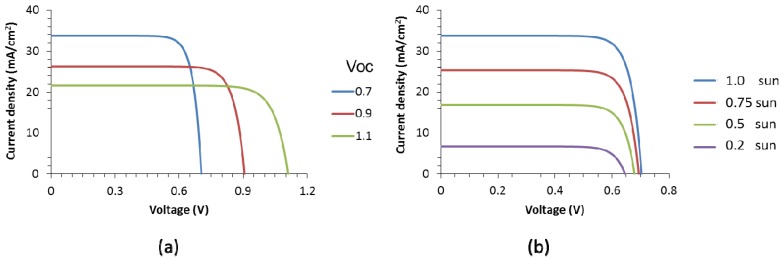
I-V characteristics used for the study (**a**) cells of 19% efficiency with different open circuit voltages (in V, see legend); and (**b**) cell with a Voc of 0.7 V for different light intensities (see legend) in which one sun is equivalent to 1000 W/m^2^).

A modest cell efficiency of 19% was chosen, as this has been reported for different thin film materials with various band gaps, which result in different open circuit voltages. Three I-V curves were chosen with an efficiency of 19% and open circuit voltages (Voc) of 0.7, 0.9 and 1.1 V, as to represent typical values for thin film CIGS, CdTe and perovskite solar cells, respectively. The curves are shown in Figure 2a. More details of the IV curves can be found in the Experimental Section.

For the curve with a Voc of 0.7 V, the illumination intensity was varied and its effect on the IV curve is shown in Figure 2b. As the light induced current density goes down, so do the Voc and the fill factor. These curves were used in the modeling to represent reference small cell without interconnection of front contact related losses.

### 2.2. Cells with a TCO Front Contact

**Figure 3:**
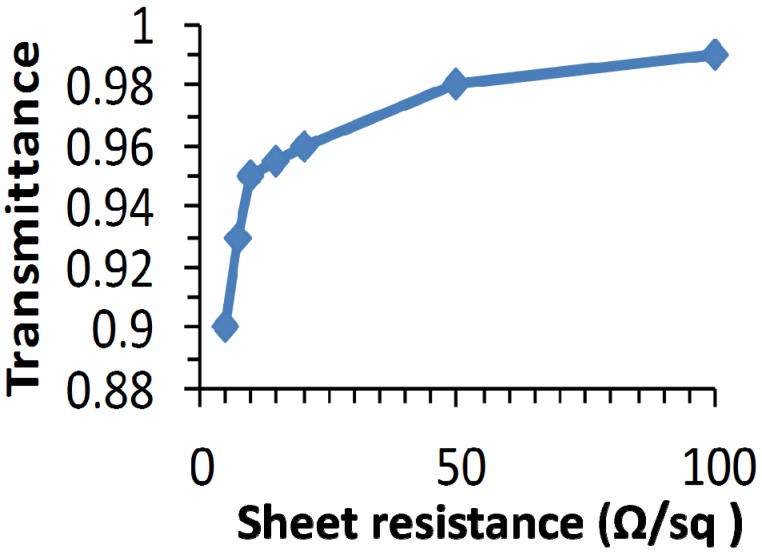
Transmittance as a function of the sheet resistance. This is used to represent TCO induced optical losses in industrially sputtered ZnO: Al material for a wavelength between 400 nm and 1100 nm and do not reflect state of the art laboratory results.

**Figure 4:**
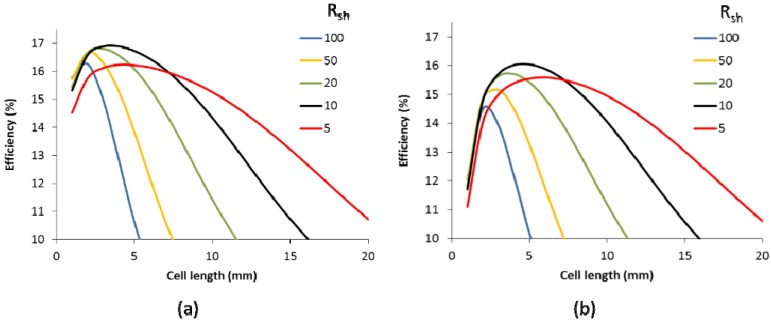
Efficiency of solar panels as a function of the individual cell length for different sheet resistances of the TCO (Rsh in Ω/sq) for a scribe width of 150 µm (**a**) and 350 µm (**b**). The cell was based on a Voc of 0.7 V.

The typical trade-off between transmittance and sheet resistance of the TCO, as used for the modeling, is shown in Figure 3. Below 10 Ω/sq, the transmittance drops with reduced sheet resistance. Figure 4a shows the efficiency as a function of the cell length for different TCO sheet resistances. The details of the TCO can be found in the Experimental Section. The cell efficiency shows a maximum with cell length. For very short cells, the optical loss related to the scribing width that is needed for isolation and interconnection is high (here taken to be 150 µm, which is near the lowest reported for CIGS [22]) compared to the total cell area. For longer cells, the efficiency drops as resistive losses become a major bottleneck. Naturally, a TCO with a lower sheet resistance allows for longer cells. However, as a lower sheet resistance goes together with a lower TCO transmittance [23,24], as shown in Figure 3, there is a trade-off and as is obvious from Figure 4a, different TCO sheet resistance have a different optimal cell length. A TCO sheet resistance of 5 Ω/sq has a long optimal cell length, but as the transmittance TCO is substantially lower than that of 10 Ω/sq, the efficiency drops from 16.9 % to 16.2%.

A maximum efficiency of 16.9% is reached for a TCO sheet resistance of 10 Ω/sq. In other words, when going from a 19% small cell to a solar panel, scribing losses and TCO related losses reduce the panel efficiency by as much as 2 absolute %. When the scribing width is enlarged to 350 µm, which is now a common value in production, the maximum obtainable cell efficiency drops to 16%, as shown in Figure 4b. This indicates the importance of careful process control and the gain that can be obtained when material removal is more carefully controlled. Moreover, the maximum cell efficiency is obtained at slightly higher cell length, but this difference is rather small. Interestingly, the difference of maximum efficiencies between the high TCOs sheet resistances is increased. This can be explained as follow: a high sheet resistance requires short cells. As the wider scribing width translates to a larger sensitivity to more narrow cells, the impact will be higher.

**Figure 5:**
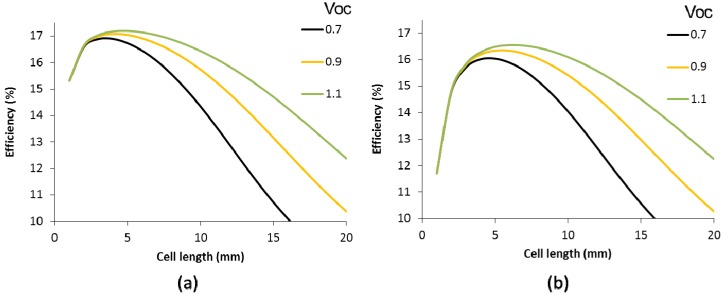
Efficiency of solar panels as a function of the individual cell length for different open circuit voltages (Voc in V) for a scribe width of 150 µm (**a**) and 350 µm (**b**). The front contact consists of a TCO of 10 Ω/sq.

Figure 5 demonstrates that high band gap materials with higher Voc translate in higher panel efficiencies, even though the small cell efficiency remains 19%. This can be explained by the fact that a higher Voc comes together with a lower short circuit density. This combination brings lower resistive losses. Moreover, lower resistive losses enable longer cells, which help to reduce the optical losses by the scribing width.

If the scribing width is increased to 350 µm, the optimal cell length increases and hence the impact of the Voc on the maximum efficiency, as shown in Figure 5b. In other words, high Voc cells are less sensitive to scribing width than cells with a low Voc. Therefore, the absorber material not only has an impact on the maximum cell efficiency, but also on the cell layout.

### 2.3. Cells with Metallic Grid

**Figure 6:**
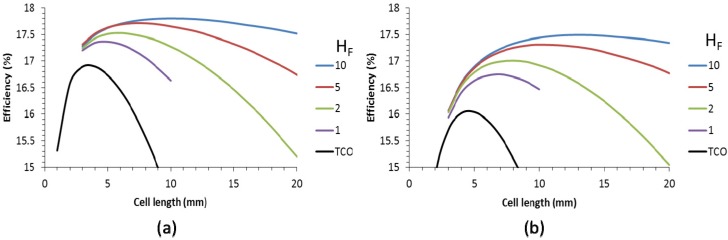
Efficiency of solar panels as function of the individual cell length for TCO-plus-grid front contact with different finger heights (H_F_, in µm) for a scribe width of 150 µm (**a**) and 350 µm (**b**). The cell was based on a Voc of 0.7 V and the finger width is 20 µm. The TCO in the legend refers to calculations with a cell with a TCO of 10 Ω/sq.

For cells with a metallic grid on top of the TCO, it was found that a TCO of 50 Ω/sq is preferable over a large range of finger widths [16]. Therefore, Figure 6 shows the efficiency as a function of the cell length for cells with a 50 Ω/sq TCO supplied with a metallic finger grid with various finger heights (*H*_F_). We also show the values for cells with a 10 Ohm/sq TCO front contact (black line).

For a scribing with of 150 µm (see Figure 6a), the efficiency increases from just below 17% to 17.8% when a high finger grid is used. For lower finger grid, the efficiency is somewhat lower and the cell length is also smaller. Nevertheless, even for a finger height of 1 µm, the increase in efficiency is 0.5 absolute %. This gain increases when a wider scribing area of 350 µm is taken into account. This is logical, because a TCO only approach cannot accommodate as long cells as compared to TCO supplemented with a finger grid, which show optimal cell lengths that are about twice that of the TCO only configuration. Therefore, the scribe area forms a lower proportion of the total area for longer cells and scribe related losses are proportionally reduced.

A grid finger height of 10 µm could be hard to accomplish for printed lines and the data also indicate the impact of lower finger heights on the cell efficiency and the optimal cell length. On the other hand, the conductivity of the finger material used for this calculation is only 1/5 of the bulk conductivity of copper. Hence, finger material improvement can further increase the efficiency [25].

**Figure 7:**
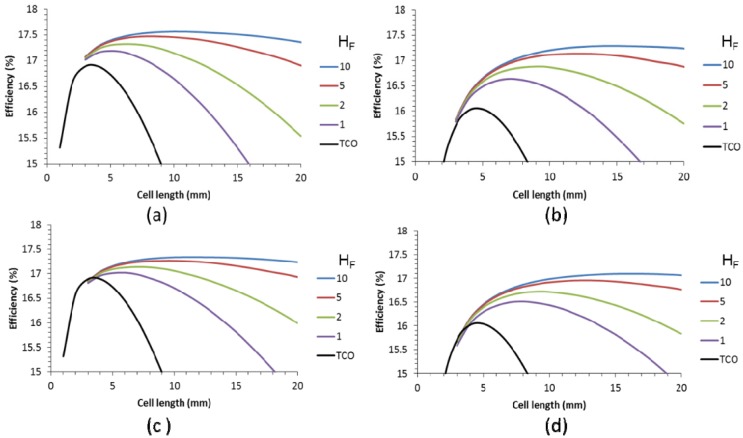
Efficiency of solar panels as a function of the individual cell length for different grid finger heights (H_F_, in µm) for a scribe width of 150 µm (**a**,**c**) and 350 µm (**b**,**d**). The finger width is 60 µm (**a**,**b**) and 100 µm (**c**,**d**). The data are based on a Voc of 0.7 V.

At present, a finger width of 20 µm is not compatible with large area printing technology. For this reason, wider fingers were also used for the calculations to assess the impact of finger width. Figure 7 shows the efficiency for cell lengths up to 20 mm, various finger heights for two different finger widths of 60 µm (Figure 7a,b) and 100 µm (Figure 7c,d). A comparison between a scribing width of 150 µm (Figure 7a,c) and 350 µm (Figure 7b,d) are also displayed.

Using a wider finger width than 20 µm decreases the efficiency benefit over the TCO only case. Nevertheless, for the presently available scribing width of 350 µm, the impact is still considerable and worth the additional manufacturing step. However, reducing the scribe width to 150 µm reduces the benefit of metallic grids.

### 2.4. Effect of Contact Resistance

**Figure 8:**
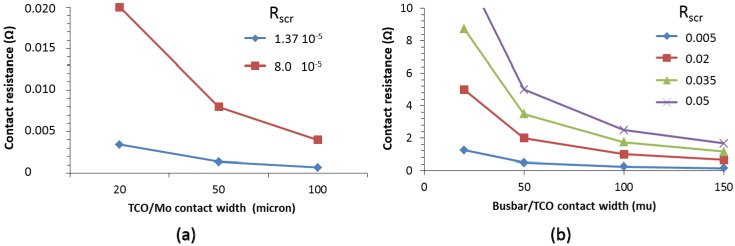
Contact resistance for a 1 cm^2^ cell as a function of the width of the contact area between the TCO and the Mo (**a**) and the metal busbar and the TCO (**b**). The legend shows the specific contact resistance (R_scr_, in Ω cm^2^). The cell length is 5 mm.

One of the topics in thin film solar cells is the effect of contact resistance, although it is seldom mentioned [26,27]. The Mo/CIGS specific contact resistance was reported to be in the order of 0.08 Ohm cm^2^ [28]. However, the specific contact resistance between TCO and Mo was found to be three orders of magnitude lower, in the range of 10^−5^ Ω cm^2^ [29]. From the specific contact resistance (R_SCR_), the contribution of the contact resistance to the overall resistance in the cell can be estimated. We calculated the contact resistance for a 1 cm^2^ cell. This was done for different widths of overlap between the TCO and the Mo, as shown in the TCO/Mo contact width in Figure 8a. For a 1 cm^2^ solar cell, typical total series resistances are between 1 and 2 ohm. For two specific contact resistances (R_scr_), the contact resistance was calculated to be less than 0.02 Ω. As this is much lower than the typical series resistance in the cell, the impact is negligible.

For printed lines, the specific contact resistance between the metal and the TCO is between 0.01 and 0.05 Ω cm^2^. At present, the factors underlying the contact resistance is under research in our group and include the ink material, the ink curing conditions and the TCO sheet resistance. The contribution of the contact resistance was calculated for a variety of various busbar/TCO overlap (contact) widths and specific contact resistances. Clearly, Figure 8b shows that the contact resistance is much higher than for the TCO/Mo case and can give a significant contribution to the overall series resistance.

**Figure 9:**
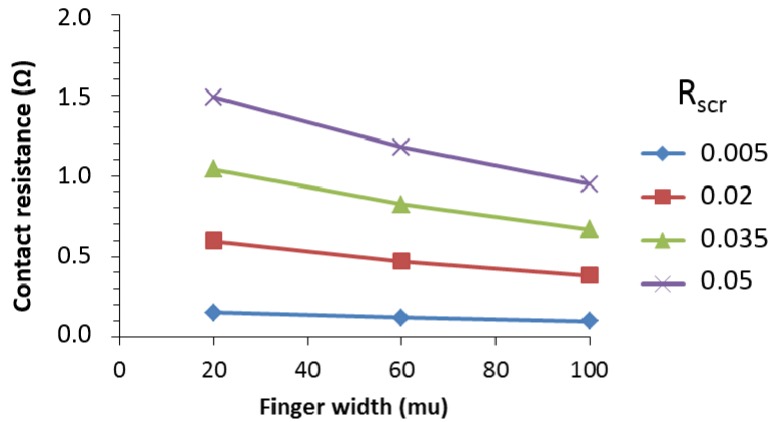
Contact resistance for a cell of 1 cm^2^ for a cell with metal interconnect and fingers as a function of the finger width for various specific contact resistances (R_scr_, in Ω cm^2^). Ω 2.5. Impact of Contact Resistance on Cell Performance.

For the case of the metallic interconnect combined with the metallic finger grid, the contact resistance was also calculated. In this case, the busbar width was taken to be 50 µm and the finger widths of 20, 60 and 100 µm were used with a finger spacing is 0.7, 1.6 and 2.1 mm, respectively. As a result of the higher contact area between the metal and the TCO, the contact resistance drops to values below 1.5 Ohm, as shown in Figure 9. This makes the system more robust against the occurrence of contact resistance between the printed metal and the TCO, although it is not negligible.

**Figure 10:**
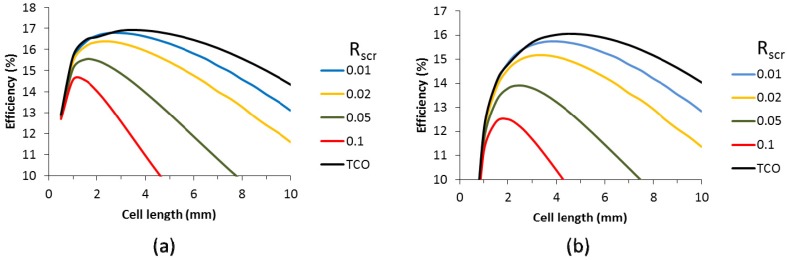
Efficiency as a function of the cell length and specific contact resistance for cells with 150 µm (**a**) and 350 µm (**b**) scribe width.

The effect of the contact resistance on the cell performance was calculated for the case without and with metallic grid. For the case without metallic grid, the data are presented in Figure 10 for a scribe with of 150 µm and 350 µm. We have used the range of specific contact resistance between 0.01 and 0.1 Ω cm^2^. The black lines indicate the case without contact resistance (TCO interconnect). A specific contact resistance of 0.01 Ω cm^2^ has only minimal impact on the cell efficiency. However, for higher specific contact resistances, the impact is larger and the efficiency drops several absolute percent for the highest specific contact resistances calculated. For a scribe width of 350 µm, the drop in efficiency is even more dramatic. This is caused by the fact that the wider scribing width induces a higher optimal cell length. The longer cells generate more current and translate into a larger effect of the series resistance. In this respect, the occurrence of contact resistance is an extra motivation to minimize the scribe width.

**Figure 11:**
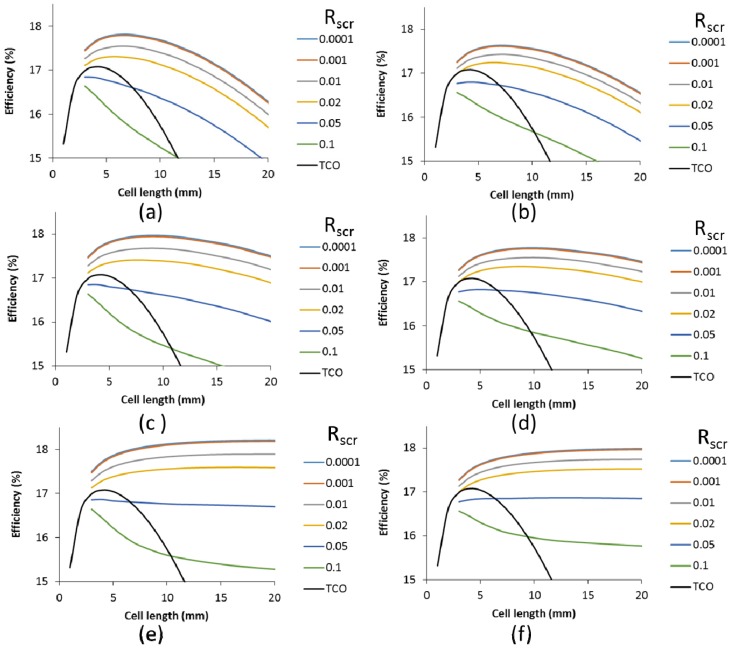
Efficiency as a function of the cell length for various specific contact resistances (see legend, R_scr_, in Ω cm^2^), and TCO (without contact resistance) for cells with finger grid width of 20 µm (**a**,**c**,**e**) and 60 µm (**b**,**d**,**f**) and a height of 2 µm (**a**,**b**), 5 µm (**c**,**d**) and 50 µm (**e**,**f**). Calculations were based on a 19% small cell.

For cells with a 50 Ω/sq TCO supplemented with a metallic finger grid, the impact of the specific contact resistance was calculated for finger widths of 20 µm and 60 µm and various finger heights, as shown in Figure 11. A scribing width of 150 µm was used. We have included lower specific contact resistances to demonstrate that extremely low values do not impact the cell efficiency. However, from a specific contact resistance of 0.01 and upward, a consistent decrease in cell efficiency and optimal cell length is seen. Above a R_scr_ of 0.02, the efficiency enhancement by the metallic grid compared to the TCO is only very small. Higher finger grids can compensate for this to some extent, but nevertheless, Figure 11 indicates that for a competitive performance of finger grids and metallic interconnect over the classic TCO interconnect, the R_scr_ should be at least 0.02 Ω cm^2^.

The effect of the R_scr_ is smaller for wider grids. This can be explained by the larger contact area between the metal and the finger. As a result, for an R_scr_ of 0.02 Ohm cm^2^ there is little difference in efficiency between the 20 µm and the 60 µm grid widths. The benefit of the lower shadow of the narrower grid finger is compensated by the higher contact resistance loss. This is independent of the grid height. Obviously, for higher grid fingers, the range of the applicable cell length increases, but the impact of the R_scr_ is similar. A longer cell increases both the contact area and the generated current and these two factors counterbalance each other.

In contrast, Figure 10 shows an increased impact of R_scr_ with cell length, as in this case, the longer cell length increases the current density, but the TCO metal contact area (busbar only) remains the same. For all cells with a metallic interconnect, the cells with a metallic grid show a higher cell efficiency (Figure 11) compared to the cells with only a TCO as the front contact for similar R_scr_ (Figure 10).

### 2.6. Influence of Illumination Power

**Figure 12:**
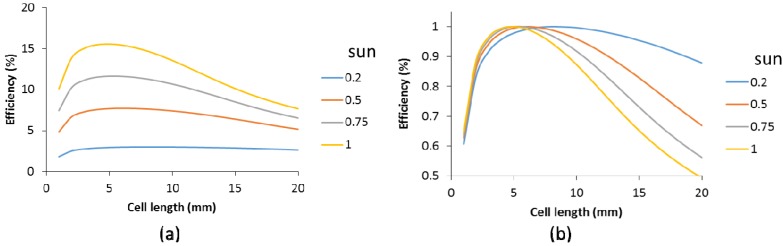
Efficiency as function of the cell length for different light intensities (see legend in sun units, whereby one sun is 1000 W/m^2^): (**a**) calculated values; (**b**) normalized values.

Solar panels and solar cells are tested and certified at an illumination power of 1000 W/m^2^ (also denoted as one sun). Therefore, the panel configuration is usually optimized for this high intensity. However, in northwest Europe, this high power is seldom reached. In real life, much of the power generated by solar panels is actually around an illumination power of 500 W/m^2^. For cells without a metallic grid, the influence of the illumination power was investigated with variation of the cell length, as shown in Figure 12a. Seemingly, as the illumination power decreases, the impact of the cell length drops. However, when these data are normalized, as shown in Figure 12b, it is seen that the relative power is merely shifted toward somewhat higher cell lengths and the impact is reduced for longer cells. Nevertheless, down to an illumination power of 0.5 suns, the cell length remains a critical part of the configuration optimization.

**Figure 13:**
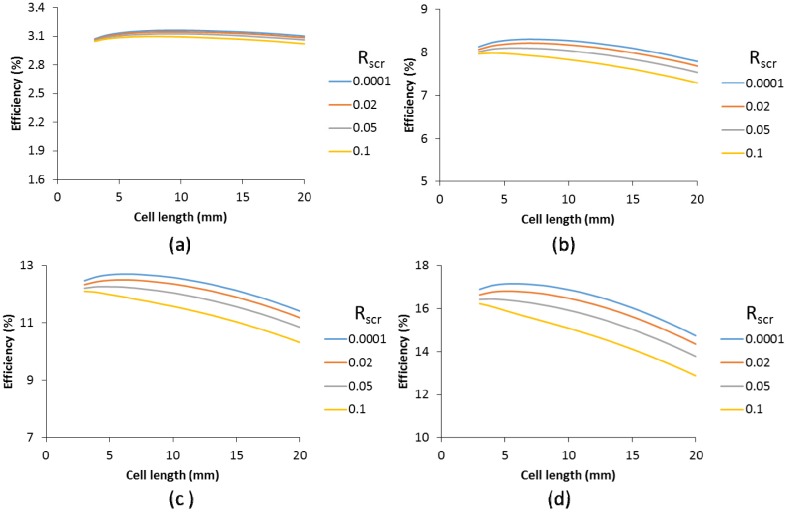
Efficiency as function of the cell length for various specific contact resistances (R_scr_, in Ohm cm^2^) for light intensity of: 0.2 sun (**a**); 0.5 sun (**b**); 0.75 sun (**c**); and 1 sun (**d**).

For cells with a finger grid, the cell efficiency seems to become less affected by the cell length, as shown in Figure 13, which shows efficiency as function of the cell length for illumination powers from 0.2 to 1 sun in Figure 13a–d. Note that for each graph, the minimum value on the x-axis is about half of the maximum value to facilitate comparison with Figure 12b. 

An additional observation is that lower illumination intensity reduces the impact of the specific contact resistance. In other words, the expected impact of the specific contact resistance for cells with metallic grids on the yearly yield of a solar panel is less than posed in Figure 10, which was based on an illumination power of one sun. Although this suggests that the R_scr_ is a less severe bottleneck, an R_scr_ below 0.02 Ω cm^2^ is still highly recommended.

## 3. Experimental Section

For the TCO only case, we have used five TCOs with various sheet resistances. The transmittance increases with sheet resistance, and this depends on the specific TCO and deposition method used. We have used the data shown in Figure 3 to represent the additional optical loss by the TCO and the values are aimed to reflect industrial application methods.

For the case of TCO plus metallic grid, a wide range of TCO values were calculated and we present here only data for a TCO sheet resistance of 50 Ω/sq. This work only discusses a finger grid configuration, because in previous work, it was determined that this is the most effective grid design for monolithically integrated thin film PV [24]. The material characteristics of the metal in the model consisted of a conductivity of 1/5 of the bulk conductivity of copper to represent a moderate quality conductive ink [30]. The specific contact resistance was varied between 0.0001 and 0.1.

The modeling was performed in Comsol and the data weres further processed in Matlab. As input, the single diode description of a solar cell with a maximum efficiency of 19% was used with the following equation: *J* = A – B * (e^−C*V^ -1), where *J* is the current density (A/m^2^) and V is voltage (V). The constants A, B and C are given in Table 1. In the model, voltage between 0 and Voc give the curves presented in Figure 2a. The constants are chosen in such a way that the maximum efficiency is 19% and the fill factor is 80%. Table 1 also shows the voltage and current density (Vmpp and Impp, respectively) at which this 19% is obtained.

**Table 1:** Constants used for the IV curves on which the modeling was based.

Material	Voc (V)	A	B	C	Vmpp (V)	Impp (A/m^2^)
CIGS	0.7	337.5	1.0 × 10^−6^	28.1	0.6	318.5
CdTe	0.9	262.7	1.0 × 10^−6^	21.55	0.77	248.1
Perovskite	1.1	215.7	1.5 × 10^−6^	17.05	0.94	203.3

## 4. Conclusions

The impact of the front contact design and interconnection material options were calculated for thin film solar cells. This includes many factors and variation of the TCO sheet resistance, scribing area, cell length, finger dimensions, contact resistance and illumination power were assessed. Metallic grids have a benefit in terms of higher solar cell efficiency and this also enables longer cell lengths. However, contact resistance between the metal and the TCO material can reduce this benefit somewhat.
